# The injectable contraceptives depot medroxyprogesterone acetate and norethisterone enanthate substantially and differentially decrease testosterone and sex hormone binding globulin levels: A secondary study from the WHICH randomized clinical trial

**DOI:** 10.1371/journal.pone.0307736

**Published:** 2024-08-23

**Authors:** Chanel Avenant, Mandisa Singata-Madliki, Alexis J. Bick, Donita Africander, Yusentha Balakrishna, Karl-Heinz Storbeck, Johnson M. Moliki, Sigcinile Dlamini, Salndave Skosana, Jenni Smit, Mags Beksinska, Ivana Beesham, Ishen Seocharan, Joanne Batting, George J. Hofmeyr, Janet P. Hapgood

**Affiliations:** 1 Department of Molecular and Cell Biology, University of Cape Town, Cape Town, South Africa; 2 Effective Care Research Unit, Eastern Cape Department of Health/Universities of the Witwatersrand and Fort Hare, East London, South Africa; 3 Department of Biochemistry, Stellenbosch University, Stellenbosch, South Africa; 4 Biostatistics Research Unit, South African Medical Research Council, Durban, South Africa; 5 Wits MRU (MatCH Research Unit), Department of Obstetrics and Gynecology, Faculty of Health Sciences, University of the Witwatersrand, Durban, South Africa; 6 Walter Sisulu University, East London, South Africa; 7 Department of Obstetrics and Gynecology, University of Botswana, Gaborone, Botswana; 8 Institute of Infectious Disease and Molecular Medicine, University of Cape Town, Cape Town, South Africa; National Institute of Allergy and Infectious Diseases, UNITED STATES OF AMERICA

## Abstract

HIV acquisition risk with norethisterone (NET) enanthate (NET-EN) is reportedly less than for depo-medroxyprogesterone acetate intramuscular (DMPA-IM). We investigated the effects of these progestin-only injectable contraceptives on serum testosterone and sex hormone binding globulin (SHBG) levels, since these may play a role in sexual behavior and HIV acquisition. The open-label WHICH clinical trial, conducted at two sites in South Africa from 2018–2019, randomized HIV-negative women aged 18–40 years to 150 mg DMPA-IM 12-weekly (n = 262) or 200 mg NET-EN 8-weekly (n = 259). We measured testosterone by UHPLC-MS/MS and SHBG by immunoassay in matched pairs of serum samples collected at baseline (D0) and at peak serum progestin levels at 25 weeks post initiation (25W) (n = 214–218 pairs). Both contraceptives substantially decreased, from D0 to 25W, the total testosterone [DMPA-IM D0 0.560, 25W 0.423 nmol/L, -24.3% (p < 0.0001); NET-EN D0 0.551, 25W 0.253 nmol/L, -54.1%, (p < 0.0001)], SHBG [DMPA-IM D0 45.0, 25W 32.7 nmol/L, -29.8% (p < 0.0001); NET-EN D0 50.2, 25W 17.6 nmol/L, -65.1% (p < 0.0001)], and calculated free testosterone levels [DMPA-IM D0 6.87, 25W 5.38 pmol/L, -17.2% (p = 0.0371); NET-EN D0 6.00, 25W 3.70, -40.0% (p < 0.0001)]. After adjusting for change from D0, the total testosterone, SHBG and calculated free testosterone levels were significantly higher for DMPA-IM than NET-EN (64.9%, p < 0.0001; 101.2%, p < 0.0001; and 38.0%, p = 0.0120, respectively). The substantial and differential decrease in testosterone and SHBG levels does not explain our previous finding of no detected decrease in risky sexual behavior or sexual function for DMPA-IM or NET-EN users from D0 to 25W. Medroxyprogesterone (MPA) and NET are androgenic and are both present in molar excess over testosterone and SHBG concentrations at 25W. Any within or between contraceptive group androgenic effects on behavior in the brain are likely dominated by the androgenic activities of MPA and NET and not by the decreased endogenous testosterone levels. The clinical trial was registered with the Pan African Clinical Trials Registry (PACTR 202009758229976).

## Introduction

Progestin-only injectable contraceptives, mainly depo-medroxyprogesterone acetate intramuscular (DMPA-IM), are the most common contraceptive methods in sub-Saharan Africa [1–3], which has a high incidence and prevalence of HIV, particularly among young women and girls [4]. Meta-analyses of higher quality observational clinical data reported a significant 40–50% increased risk of HIV acquisition with DMPA-IM compared to no hormonal contraception, unlike for limited data on NET-EN [5, 6]. Head-to-head comparisons of HIV risk among women using DMPA-IM versus NET-EN indicated a potential 32–41% increase in HIV risk for DMPA-IM users versus NET-EN users [5, 7, 8]. The Evidence for Contraceptive Options and HIV Outcomes (ECHO) randomized trial comparing DMPA-IM, the copper intra uterine device (IUD) and Jadelle, a levonorgestrel (LNG)-containing implant, did not detect a significant difference in HIV acquisition risk of 50% or more between these methods [9]. However, the ECHO trial data do not inform on the risk for HIV infection for DMPA-IM compared to NET-EN, or for DMPA-IM compared to no contraception or irregular use of condoms [9]. Obtaining robust data on the effects of DMPA-IM and NET-EN on factors that potentially affect risk for HIV acquisition is important for understanding their risks and benefits and the biological mechanisms thereof. The Women’s Health, Injectable Contraception and HIV (WHICH) trial, an open-label clinical trial that randomized women to DMPA-IM or NET-EN, investigated differences in hormonal, psychological, behavioral, menstrual and immune effects within and between the two contraceptives. While both contraceptives substantially and similarly reduced estradiol to postmenopausal levels one week after the 6-month injection, the data suggested more sexual exposure to HIV with DMPA-IM than NET-EN [10].

Levels of endogenous sex steroid hormones are likely to play a role in multiple physiological pathways and health outcomes, including susceptibility to sexually transmitted infections and HIV, immune function and sexual behavior [11]. While estradiol is known to be protective against HIV acquisition in the female genital tract [11], the role of androgens [12, 13] in HIV acquisition is unknown. Circulating levels of sex hormones reportedly modify cellular morphology in the brain [14] and influence higher brain functions such as cognition, memory and mood [15]. Indeed, decreased testosterone levels are associated with several undesired effects such as increased headaches, mood changes, and reduced sexual desire and libido [16]. Thus, decreased testosterone levels may decrease libido resulting in less exposure to HIV. Evidence on the effects of libido is, however, contentious as some women on combined oral contraception (COC) had decreased testosterone levels without a decrease in libido [17]. It is also unclear whether sexual function in women is associated with endogenous androgen concentrations due to insufficient robust data and uncertainty relating to the sensitivity and specificity of androgen quantification assays in some studies [18]. In addition, hormonal contraception is also associated with several androgenic effects including acne, hirsutism, weight gain, androgenic alopecia, unfavourable lipid profiles, and diabetes [19–23]. DMPA-IM has been linked to increased incidence of type 2 diabetes, oily skin and acne in women [20, 22–24], while very little information is available on the androgenic effects of NET-EN in women. Furthermore, it is unknown to what extent any androgenic effects may be attributed to the known androgenic activity of MPA and NET via the androgen receptor (AR) *in vitro* [25].

Testosterone is one of the major androgens in the serum of premenopausal women [26, 27]. However, clinical studies investigating the effects of progestin-only contraceptives on testosterone levels are limited, and the available studies mainly assessed effects in women using COCs which result in reduced total serum testosterone concentrations (reviewed in [12]). Earlier studies showed decreased serum testosterone levels in postmenopausal breast cancer patients after administration of oral MPA [28]. Similarly, it has been shown that subcutaneous DMPA (DMPA-SC) [29] and the LNG implant [30] both decrease testosterone levels in premenopausal women. A decrease in testosterone was also detected in premenopausal women (3–4 women) and in a transgender population [31] administered DMPA-IM, while a significant decrease in testosterone was not detected in postpartum women administered NET-EN [32]. To understand the clinical significance of changes in testosterone levels in women, it is important to have accurate data on the effects of progestins not only on total testosterone levels, but also that of free testosterone and sex hormone binding globulin (SHBG) levels. Total testosterone includes both the biologically inactive, circulating, SHBG-bound testosterone, as well as the biologically active testosterone circulating either free (not bound to plasma protein) or that weakly bound to albumin [33]. Studies to date suggest that the effects of progestins on testosterone and SHBG are dependent on the type of contraceptive. For instance, ethinyl estradiol combined with either LNG or drospirenone (DRSP) reduced total and free testosterone levels, but increased SHBG levels [34]. This increase in SHBG levels is likely due to ethinyl estradiol as it is known to increase hepatic SHBG production (reviewed in [35]). In contrast, the progestin-only injectable DMPA-SC is associated with a significant decrease in total testosterone and SHBG, but not in free testosterone levels [29]. To our knowledge, only two other non-comparative studies have shown that the injectable contraceptives DMPA-IM [36] and NET-EN [37] decrease SHBG levels.

There is a lack of robust data on the effects of DMPA-IM and NET-EN on testosterone and SHBG levels, as well as their relative effects. Furthermore, no data are available for the effects of these contraceptives at peak serum progestin levels. In this study, we compared the serum levels of testosterone and SHBG, as well as changes in the levels of total and calculated free testosterone and SHBG, within and between two arms of the WHICH trial randomizing women to DMPA-IM or NET-EN, at peak progestin levels.

## Methods

### Primary study, ethics and biosafety

This study is a secondary study from the open-label randomized WHICH clinical trial. The primary aims of the trial were estradiol levels and menstrual, psychological and behavioral measures relevant to HIV risk. The WHICH study protocol and primary study have been reported elsewhere [10]. The study was registered retrospectively with the Pan African Clinical Trials Registry (PACTR 202009758229976 https://pactr.samrc.ac.za/Search.aspx). All women provided informed, written consent to authorize study participation and storage of samples. The study adhered to the ethical principles outlined in the Declaration of Helsinki (World Medical Association, 2011) and the Constitution of the Republic of South Africa (Bill of Rights). Ethical approval for the secondary study conducted at the University of Cape Town (UCT) was obtained from the UCT Faculty of Health Sciences Human Research Ethics Committee (HREC REF no. 664/2018). The authors did not have access to information that could identify individual participants during or after data collection.

### Study design and sample collection

Briefly, HIV-negative young women (18–40 years) seeking contraception at the East London and Mdantsane public health clinics and hospitals (Frere and Cecilia Makiwane Hospitals), South Africa (331 participants), and the research site of the MatCH Research Unit (MRU), University of the Witwatersrand, based in Durban, KwaZulu-Natal, South Africa (189 participants) were randomized to 150 mg DMPA-IM 12-weekly or 200 mg NET-EN 8-weekly. Exclusion criteria were participants who received DMPA-IM in the previous 6 months or NET-EN in the previous 4 months, were living with HIV, or were using or intending to use medication which might have interfered with biological measurements such as steroids or drugs affecting renal function such as pre-exposure prophylaxis drugs (for HIV). Participants were recruited and followed from 5 November 2018 to 30 November 2019. We screened 546 and randomized 521 women to DMPA-IM (262) and NET-EN (259). A total of 86.9% (n = 453) completed a 25-week study visit with a similar number completing in both method groups.

Blood samples were collected at baseline (D0) and at 25 weeks (25W), i.e. about one week after the 6-month NET-EN (the 4^th^ NET-EN injection) or DMPA-IM (the 3^rd^ DMPA-IM) injection, and serum was separated and stored at -80°C.

### Total testosterone measurements

These measurements were performed between 2 January 2020 and 31 December 2022. Testosterone was quantified by ultra-high performance liquid tandem mass spectrometry (UHPLC-MS/MS) on stored serum samples from WHICH study participants at D0 and 25W, as described in S1 Appendix. Testosterone data were obtained for 214 and 215 participants, at D0 and 25W respectively, from the DMPA-IM arm, with D0 data for one participant being absent for technical reasons. Testosterone data were obtained for 219 and 218 participants at D0 and 25W, respectively from the NET-EN arm. Accuracy (% BIAS) and precision (% CV) were both less than 15% at all concentrations tested (0.05, 0.1, 0.5, 5 and 50 ng/mL) (S1 Table in S1 File). Recovery (% Extraction efficiency) and matrix effects were both within acceptable limits (S1 Table in S1 File). The limit of detection (LOD) and lower limit of quantification (LLOQ) for testosterone were 0.0250 ng/mL (0.0870 nmol/L) and 0.0500 ng/mL (0.173 nmol/L), respectively, while the upper limit of quantification (ULOQ) was 50.0 ng/mL (173.37 nmol/L) (S2 Table in S1 File). A linear calibration curve was obtained between the LLOQ and ULOQ (R^2^ > 0.996). All researchers performing the assays were blinded.

### SHBG measurements and calculation of free testosterone

These measurements were performed between 2 January 2020 and 31 December 2020. SHBG was measured on stored serum samples from WHICH study participants at D0 and 25W by means of chemiluminescent microparticle immunoassay (CMIA) (Abbott Laboratories) (sensitivity level of quantification (LOQ) 0.02 nmol/L; no detectable cross-reactivity). All researchers performing the assays were blinded. Free testosterone was calculated according to the method of Vermeulen et al. [38]. SHBG data were obtained for 217 and 216 participants, at D0 and 25W respectively, from the DMPA-IM arm, with 25W data for one participant being absent for technical reasons. SHBG data were obtained for 219 and 218 participants at D0 and 25W, respectively, from the NET-EN arm, with 25W data for one participant being absent for technical reasons. Free testosterone was calculated for all those participants for whom SHBG and testosterone data was available (214 and 215 for D0 and 25W, respectively in DMPA-IM arm; 219 and 217 for D0 and 25W, respectively, for NET-EN arm).

### Data analysis

UHPLC-MS/MS data collection and analysis were performed using MassLynx 4.2 (Waters Corporation). The ratio of the analyte peak area to internal standard peak area was determined for all the calibration curve samples, internal quality controls (IQCs) and serum samples. Testosterone values below the LLOQ, but above the LOD were assigned 0.5 x LLOQ (n = 16 D0; n = 30 25W), while values lower than LOD were assigned as 0.000 (n = 5 25W). For SHBG all the values were above the level of quantification (LOQ) (0.02 nmol/L).

We performed a modified intention-to-treat (mITT) analysis on the whole cohort and a per protocol (PP) analysis on a subgroup of the whole cohort. To obtain a subgroup for the PP analysis, we used UHPLC-MS/MS data obtained for study (MPA and NET) and non-study (LNG, nestorone, etonogestrel and gestodene) progestin levels at D0 and 25W in donor-matched serum samples [39]. For the testosterone and SHBG subgroup PP analysis, we excluded results from all women (104/436 participants, or 23.8%) that had any non-study serum progestin at concentrations ≥ 1.5 nM, at either D0 or 25W, from the mITT group. In addition to the above-mentioned non-study progestins, for the PP analysis we also excluded women in the DMPA-IM arm that had NET concentrations greater than 1.5 nM, at either D0 or 25W, as well as women in the NET-EN arm that had MPA concentrations higher than 1.5 nM, at either D0 or 25W. In total 23.8% (104/346 participants) of the women were excluded in the PP analysis.

Results in Tables 2–4 and S3 Table in S1 File were analysed using Stata version 16 (College Station, TX: StataCorp LLC). For total testosterone and SHBG, D0 and 25W values were nmol/L, while pmol/L values for free testosterone were used. Shapiro-Wilks normality tests indicated that all hormonal data were not normally distributed, and hence data are expressed as median with interquartile range (IQR) (Table 2 and S3 Table in S1 File). A mixed-effects linear regression model was fitted for each of the (natural) log-transformed outcomes (total testosterone, SHBG, free testosterone). Random effects to account for repeated measures within participants and to account for clustering by site were included in the model. A model coefficient, *β*, on the log-scale can be back-transformed using *e*^*β*^. To facilitate the interpretation of the results, we report the percentage changes on the original scale, calculated as (*e*^*β*^ – 1) × 100%. Thus, the mean differences between D0 and 25W as well as between allocated arms are presented as percentages (back-transformed coefficients) with 95% confidence intervals (CIs) (Tables 3 and 4). Fig 2 and S1 Fig in S1 File were generated using GraphPad Prism 9.31 from GraphPad Software, Inc. (La Jolla California, USA), while statistical differences determined by mixed-effects linear regression model, as mentioned above, are shown. All results were considered significant for p < 0.05.

## Results

### Primary data and baseline characteristics

**Fig 1:**
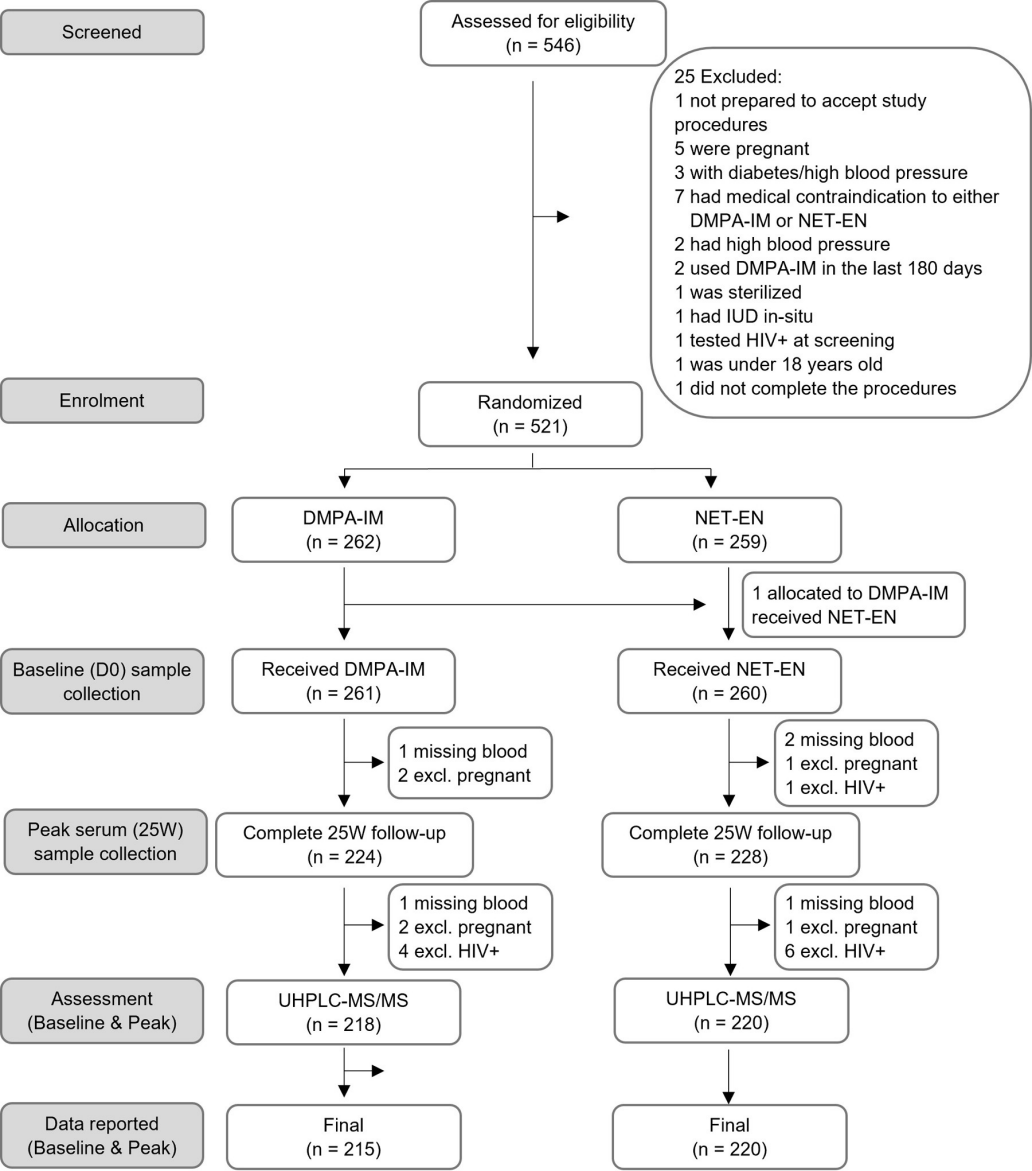
Trial profile.

Of 521 participants enrolled, results are reported for all matched serum samples available from the whole cohort for 435 (83%) participants both at baseline and at peak (mITT analysis). The trial profile is shown in Fig 1.The excluded participants in the mITT analysis include 11 (2%) that became HIV positive, 6 (1%) that became pregnant, 65 (12%) that were lost to follow up (i.e., those that did not provide a 25W blood sample) and 5 participants that had a missing blood result for either SHBG or for testosterone, due to technical problems with the sample.

Baseline data is shown in Table 1.

**Table 1:** Baseline characteristics of women by randomization method^$^ (mITT analysis).

	Baseline (D0)			
	DMPA-IM		NET-EN	
		n		n
**Age, years: Mean (SD)**	25 (4.8)	217	24.9 (4.7)	219
**Ethnicity**		217		219
Xhosa	145 (66.8)		148 (67.6)	
Zulu	67 (30.9)		71 (32.4)	
Mixed race	1 (0.5)		0 (0.0)	
Other African ethnicity	4 (1.8)		0 (0.0)	
**Previous use of method** ^**#**^		217		219
DMPA-IM	161 (74.2)		160 (73.1)	
NET-EN	69 (31.8)		65 (29.7)	
**Marital status**		217		219
Single	211 (97.2)		213 (97.3)	
Married	6 (2.8)		6 (2.7)	
**Highest level of education**		217		219
Primary school, complete	2 (0.9)		4 (1.8)	
High school, not complete	88 (40.6)		76 (34.7)	
High school, complete	82 (37.8)		95 (43.4)	
Post high school education	45 (20.7)		44 (20.1)	
**Source of income**		217		219
Unemployed	183 (84.3)		192 (87.7)	
Employed	34 (15.7)		27 (12.3)	

^$^ Unless indicated otherwise, values represent n-value (%)

^#^Prior to exclusion period, numbers given are for those that responded, and in brackets are % of those that responded. Note that some participants reported using both contraceptive methods prior to the exclusion period.

### DMPA-IM and NET-EN decrease total and free testosterone and SHBG concentrations

**Fig 2:**
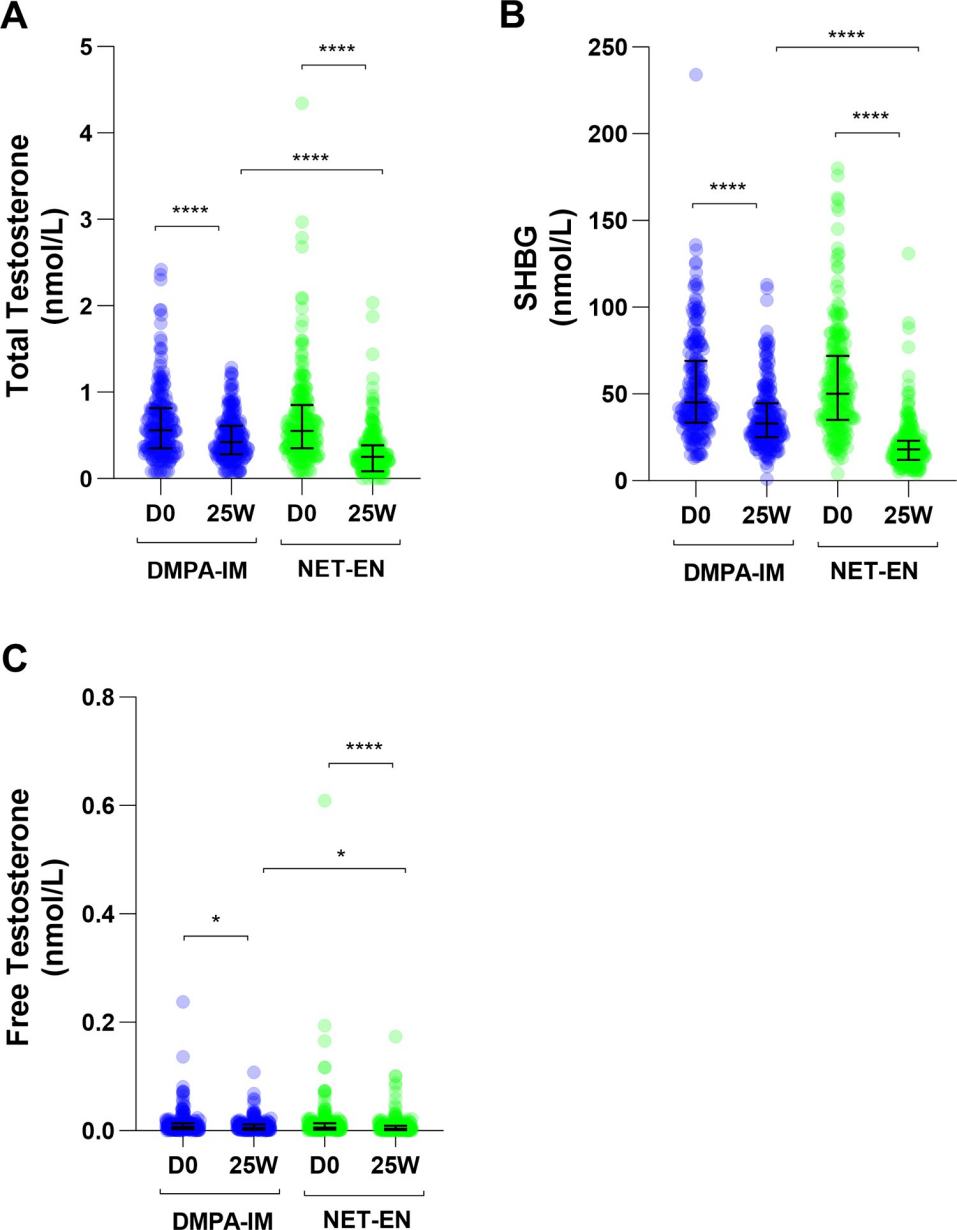
Total testosterone (nmol/L), SHBG (nmol/L) and free testosterone (nmol/L), outcomes at baseline and 25 weeks (mITT analysis). Graphs indicate median with interquartile range (IQR). Significant differences were calculated by mixed effects linear regression, accounting for repeated measurements per participant and clustering by site and are indicated by asterisks where * and **** represent p<0.05 and p<0.0001, respectively.

At baseline, the median total testosterone levels in the DMPA-IM and NET-EN arms were 0.560 nmol/L and 0.551 nmol/L, respectively (Table 2 and Fig 2). Both DMPA-IM and NET-EN significantly decreased total testosterone levels from D0 to 25W by 24.3% (p < 0.0001) and 54.1% (p < 0.0001), respectively (Table 3), with median concentrations at 25W in the DMPA-IM and NET-EN arms being 0.423 nmol/L and 0.253 nmol/L, respectively (Table 2). A significant difference in total testosterone levels between arms was detected at 25W with DMPA users having a 60.3% higher testosterone level than NET-EN users (p < 0.0001) (Table 4). No significant difference in total testosterone levels between arms was detected at D0 (Table 4). A significant difference was detected in total testosterone levels at 25W between arms, after adjusting for baseline, with DMPA-IM users showing a 64.9% (p < 0.0001) higher total testosterone level than NET-EN users (Table 4).

**Table 2:** Total testosterone (nmol/L), SHBG (nmol/L) and free testosterone (pmol/L) outcomes at baseline and 25 weeks (mITT analysis).

	DMPA-IM		NET-EN	
	Median (IQR)	n	Median (IQR)	n
**Total Testosterone (nmol/L)**				
D0	0.560 (0.354; 0.815)	214	0.551 (0.350; 0.853)	219
25W	0.423 (0.281; 0.610)	215	0.253 (0.086; 0.385)	218
Change (25W - D0)	-0.119 (-0.288; 0.003)		-0.291 (-0.513; -0.090)	
**SHBG (nmol/L)**				
D0	45.0 (33.6; 68.8)	217	50.2 (35.1; 72.4)	219
25W	32.7 (24.8; 44.4)	216	17.6 (12.2; 22.9)	218
Change (25W - D0)	-12.2 (-25.1; -2.3)		-32.3 (-51.5; -17.7)	
**Free Testosterone (pmol/L)**				
D0	6.87 (2.81; 13.82)	214	6.00 (2.00; 14.0)	219
25W	5.38 (2.28; 11.73)	215	3.70 (0.87; 9.33)	217
Change (25W - D0)	-0.71 (-5.48; 1.33)		-1.50 (-7.02; 1.16)	

IQR (25th and 75th Percentile)

**Table 3:** Mean percentage changes (95% CI) in total testosterone, SHBG and free testosterone (mITT analysis).

	DMPA-IM	NET-EN	DMPA-IM vs NET-EN
	Mean (95% CI)*	Mean (95% CI)*	p-value*
**Total Testosterone**			
% Change from D0	-24.3 (-30.5; -17.5)	-54.1 (-57.9; -50.0)	**<0.0001**
**SHBG**			
% Change from D0	-29.8 (-34.6; -24.7)	-65.1 (-67.5; -62.6)	**<0.0001**
**Free Testosterone**			
% Change from D0	-17.2 (-30.7; -1.1)	-40.0 (-49.8; -28.4)	**0.0120**

* estimates and p-values from mixed effects linear regression, accounting for repeated measurements per participant and clustering by site. Note, mixed effects linear regression indicated that both DMPA-IM and NET-EN significantly decreased total testosterone (p < 0.0001 for both), SHBG (p < 0.0001 for both) and free testosterone (DMPA-IM p = 0.0371; NET-EN p < 0.0001) levels from D0 to 25W.

**Table 4:** Pairwise comparisons of hormonal results for DMPA-IM vs NET-EN expressed as mean percentage differences (95% CI) (mITT analysis).

	Mean percentage difference between DMPA-IM and NET-EN at D0		Mean percentage difference between DMPA-IM and NET-EN at 25W		Mean percentage difference at 25W between DMPA-IM and NET-EN, after adjusting for baseline	
	DMPA_D0_ –NET-EN_D0_		DMPA_25W_ –NET-EN_25W_			
	Mean % (95% CI)*	p-value*	Mean % (95% CI)*	p-value*	Mean % (95% CI)*	p-value*
**Total Testosterone**	-2.9 (-13.6; 9.3)	0.6347	60.3 (42.4; 80.4)	<0.0001	64.9 (46.1; 86.1)	<0.0001
**SHBG**	-5.6 (-14.6; 4.3)	0.2556	89.9 (71.8; 109.9)	<0.0001	101.2 (82.2; 122.3)	<0.0001
**Free testosterone**	-0.2 (-21.9; 27.5)	0.9867	37.7 (7.6; 76.2)	0.0110	38.0 (7.3; 77.4)	0.0120

*estimates and p-values from mixed effects linear regression, accounting for repeated measurements per participant and clustering by site.

At baseline the median SHBG levels in the DMPA-IM and NET-EN arms were 45.0 nmol/L and 50.2 nmol/L, respectively (Table 2 and Fig 2). Both DMPA-IM and NET-EN significantly reduced SHBG levels from D0 to 25W by 29.8% (p < 0.0001) and 65.1% (p < 0.0001), respectively (Table 3), with median SHBG levels at 25W in the DMPA-IM and NET-EN arms being 32.7 nmol/L and 17.6 nmol/L, respectively (Table 2 and Fig 2). A significant difference in SHBG levels between arms was detected at 25W with DMPA users having an 89.9% higher SHBG level than NET-EN users (p < 0.0001) (Table 4). At D0 no significant difference in SHBG levels was detected between the two arms (Table 4). After adjusting for the change from D0, DMPA-IM users had 101.2% (p < 0.0001) higher SHBG levels than NET-EN users at 25W (Table 4).

Median calculated free testosterone concentrations at baseline in the DMPA-IM and NET-EN arm were 6.87 pmol/L and 6.00 pmol/L, respectively (Fig 2 and Table 2). At 25W the median free testosterone levels in the DMPA-IM and NET-EN arms were 5.38 pmol/L and 3.70 pmol/L, respectively (Table 2). Both DMPA-IM and NET-EN significantly reduced free testosterone levels from D0 to 25W by 17.2% (p = 0.0371) and 40.0% (p < 0.0001), respectively (Table 3). Additionally, a significant difference in free testosterone levels between arms was detected at 25W with DMPA users having a 37.7% higher free testosterone level than NET-EN users (p = 0.0110) (Table 4). At D0 no significant difference in free testosterone levels was detected between the two arms (Table 4). After adjusting for the change from D0, DMPA-IM users had 38.0% (p = 0.0120) higher free testosterone levels at 25W than NET-EN users (Table 4).

In a subgroup PP analysis, after excluding for non-study progestins, significant differences were detected for the same comparisons for the whole cohort (mITT analysis) compared to the subgroup (PP analysis), for total testosterone, SHBG and free testosterone concentrations (S3 Table and S1 Fig in S1 File). The baseline characteristics for the subgroup of women randomized to DMPA-IM or NET-EN used in the PP analysis are shown in S4 Table in S1 File.

## Discussion

We report for the first time on the effects of DMPA-IM and NET-EN on total and free testosterone and SHBG levels at peak progestin levels from a randomized trial. We detected substantial decreases from D0 to 25W in measured total testosterone (-24.3% and -54.1%) and SHBG levels (-29.8% and -65.1%) and calculated free testosterone levels (-17.2% and -40.0%), for DMPA-IM and NET-EN users, respectively. Whether the lower median concentrations of total testosterone at 25W could be classified as hypoandrogenic or post-menopausal, is unclear from the literature [27, 40, 41]. When comparing the mean percentage difference in change between these contraceptives from D0 to 25W, we report that DMPA-IM use results in a significantly smaller change in total testosterone, SHBG levels and free testosterone than NET-EN use.

To our knowledge there is no published literature to allow direct comparison between our results for DMPA-IM and NET-EN at peak progestin concentrations. One observational study reported values for DMPA-IM that are 27-fold greater and 2.5-fold lower [42] than the values we determined for total and free testosterone, respectively. A study among 15 women using the 104 mg DMPA-SC injection reported a significant decrease in total testosterone and SHBG, measured by immunoassay, but not for calculated free testosterone, one week after the 3-month DMPA-SC injection [29]. Their values are about 2–4-fold higher than our median total testosterone values, but comparable to our median SHBG values and about 5–6-fold lower than our values calculated for free testosterone. Very little information is available for NET-EN, but one study reported a reduction in SHBG levels five days after NET-EN injection, with levels of SHBG 2.3-fold higher than our value at 25W [37]. Possible reasons for differences between our results and other results for DMPA-IM or NET-EN include lower power due to small sample sizes, confounding factors due to non-randomization, and differences in sampling times and methods of testosterone quantification. Our use of UHPLC-MS/MS is likely to have generated more accurate and lower values for testosterone than immunoassay methods [43, 44]. Nevertheless, our results, reviewed together with limited published data [45], suggest that most progestin-only contraceptives significantly and substantially decrease SHBG levels, and that DMPA-IM decreases total and calculated free testosterone levels to a greater extent than DMPA-SC at peak serum MPA levels, while both have and similar effects on SHBG levels.

The biological, behavioral and clinical consequences of substantially decreased levels of total and free serum testosterone and SHBG for both DMPA-IM and NET-EN are difficult to interpret. When comparing effects on sexual behavior between DMPA-IM and NET-EN, we have reported more sexual activity and more risky sexual behavior and possibly more exposure to HIV with DMPA-IM than NET-EN [10]. This is consistent with a greater decrease in total testosterone levels for NET-EN relative to DMPA-IM, as testosterone may be associated with increased libido. However, while the relative effects of these contraceptives are important, the individual effects from baseline to 25W are also biologically and clinically relevant. If increased testosterone results in increased sexual activity in women, one would expect a decrease in sexual behavior within both arms. However, a substantial decrease in sexual behavior from baseline to 25W was not detected in the WHICH cohort for either contraceptive [10].

An important potential confounding factor in understanding the effects of DMPA-IM and NET-EN on androgenic effects is that both these progestins are themselves androgenic. They have similar binding affinities for and potencies via the AR *in vitro* when compared head-to-head to each other and dihydrotestosterone [25, 46], although one study reported that MPA is less potent than testosterone and dihydrotestosterone [47]. While some studies have reported that NET has more androgenic activity than MPA in animal pre-clinical models [48], there is no robust data on androgenic activity of DMPA-IM and NET-EN in women. It is not possible to extrapolate these *in vitro* and animal data directly to relative androgenic activities in women due to multiple confounding factors, including metabolism, cross-talk with other pathways and species-, gene- and cell-specific effects. We have reported that the medium peak serum concentrations for MPA and NET in DMPA-IM and NET-EN users are 6.6 and 14 nmol/L, respectively, in these WHICH trial samples [39]. Notably, these concentrations are much higher than those for endogenous total or calculated free testosterone [DMPA-IM 0.423 nmol/L and 5.38 pmol/L; NET-EN 0.253 nmol/L and 3.70 pmol/L, respectively] as well as those of estradiol (25W DMPA-IM 77 pmol/L; 25W NET-EN 70 pmol/L) [10]. Thus, it is possible that any androgenic effects of DMPA-IM and NET-EN are dominated by the androgenic properties of MPA and NET themselves, rather than the relatively very low but differential levels of testosterone reported here, and postmenopausal but similar levels of estradiol for both contraceptives [10].

One potential confounding factor in our randomized study is misreporting of DMPA-IM or NET-EN use, or use of contraceptives containing estrogens such as COCs, before and/or during the trial. We and others have reported that it is common for women not to self-report non-study progestin use before initiation and during clinical trials on contraception [39, 49]. We performed a PP analysis of the testosterone and SHBG data on a subgroup of participants after excluding those with concentrations of non-study progestins above 1.5 nM at either D0 or 25W. These PP analyses resulted in the same significant differences compared to the mITT results, and comparable median values for total and free testosterone and SHBG, suggesting that non-study progestin use did not introduce significant bias to these values. Our testosterone and SHBG findings are unlikely to be confounded by differences in baseline characteristics between contraceptive arms, given the stringent randomization process.

## Conclusions

Total and free testosterone and SHBG potentially affect multiple physiological pathways and clinical outcomes. Our published findings of increased risky sexual behavior for DMPA-IM relative to NET-EN users are consistent with the differential decrease in total endogenous testosterone levels. However, our lack of detection of a general decrease in risky sexual behavior from D0 to 25W for either contraceptive is not consistent with a substantial decrease in endogenous testosterone levels being the major determinant of changes in sexual behavior. Taken together, it is likely that progestins themselves are the major determinants of androgenic effects, including on sexual behavior in the brain, that potentially affect HIV acquisition risk for DMPA-IM and NET-EN, as well as their relative effects. Understanding these complex mechanisms requires more research.

## Supporting information

S1 File S1 Fig and S1 to S4 Tables. (DOCX)

S1 Appendix Appendix 1 testosterone quantification. (DOCX)

## References

[1] National Department of Health (NDoH), Statistics South Africa (Stats SA), South African Medical Research Council (SAMRC), ICF. South Africa Demographic and Health Survey 2016. Pretoria, South Africa and Rockville, Maryland, USA: NDoH, Stats SA, SAMRC, and ICF; 2019. Available from: http://dhsprogram.com/pubs/pdf/FR337/FR337.pdf.

[2] UNAIDS. Joint United Nations Programme on HIV/AIDS. 2020 Global AIDS Update ‐ Seizing the Moment ‐ Tackling entrenched inequalities to end epidemics. Geneva, Switzerland: UNAIDS; 2020 6 July 2020. Available from: https://www.unaids.org/en/resources/documents/2020/global-aids-report.

[3] United Nations Department of Economic and Social Affairs Population Division. Contraceptive Use by Method 2019: Data Booklet (ST/ESA/SER.A/435). 2019. Available from: https://digitallibrary.un.org/record/3849735?ln=en.

[4] UNAIDS. Joint United Nations Programme on HIV/AIDS. UNAIDS DATA 2021. Geneva, Switzerland: UNAIDS; 2021. Available from: https://www.unaids.org/en/resources/documents/2021/2021_unaids_data.

[5] Morrison CS, Chen PL, Kwok C, Baeten JM, Brown J, Crook AM, et al. Hormonal contraception and the risk of HIV acquisition: an individual participant data meta-analysis. PLoS Med. 2015; 12(1):e1001778. doi: 10.1371/journal.pmed.1001778 25612136 PMC4303292

[6] Polis CB, Curtis KM, Hannaford PC, Phillips SJ, Chipato T, Kiarie JN, et al. An updated systematic review of epidemiological evidence on hormonal contraceptive methods and HIV acquisition in women. AIDS. 2016; 30(17):2665–83. doi: 10.1097/QAD.0000000000001228 27500670 PMC5106090

[7] Heffron R, Achilles SL, Dorflinger LJ, Hapgood JP, Kiarie J, Polis CB, et al. Pharmacokinetic, biologic and epidemiologic differences in MPA- and NET-based progestin-only injectable contraceptives relative to the potential impact on HIV acquisition in women. Contraception. 2019; 99(4):199–204. doi: 10.1016/j.contraception.2018.12.001 30576636 PMC6467541

[8] Noguchi LM, Richardson BA, Baeten JM, Hillier SL, Balkus JE, Chirenje ZM, et al. Risk of HIV-1 acquisition among women who use different types of injectable progestin contraception in South Africa: a prospective cohort study. Lancet HIV. 2015; 2(7):e279–87. doi: 10.1016/S2352-3018(15)00058-2 26155597 PMC4491329

[9] Evidence for Contraceptive Options and HIV Outcomes (ECHO) Trial Consortium. HIV incidence among women using intramuscular depot medroxyprogesterone acetate, a copper intrauterine device, or a levonorgestrel implant for contraception: a randomised, multicentre, open-label trial. Lancet. 2019; 394(10195):303–13. 31204114 10.1016/S0140-6736(19)31288-7 PMC6675739

[10] Singata-Madliki M, Smit J, Beksinska M, Balakrishna Y, Avenant C, Beesham I, et al. Effects of injectable contraception with depot medroxyprogesterone acetate or norethisterone enanthate on estradiol levels and menstrual, psychological and behavioral measures relevant to HIV risk: the WHICH randomized trial. PLoS One. 2024; 19(3): e0295764. doi: 10.1371/journal.pone.0295764 38530848 PMC10965066

[11] Wira CR, Rodriguez-Garcia M, Patel MV. The role of sex hormones in immune protection of the female reproductive tract. Nat Rev Immunol. 2015; 15(4):217–30. doi: 10.1038/nri3819 25743222 PMC4716657

[12] Louw-du Toit R, Storbeck KH, Cartwright M, Cabral A, Africander D. Progestins used in endocrine therapy and the implications for the biosynthesis and metabolism of endogenous steroid hormones. Mol Cell Endocrinol. 2017; 441:31–45. doi: 10.1016/j.mce.2016.09.004 27616670

[13] Simpson ER, Clyne C, Rubin G, Boon WC, Robertson K, Britt K, et al. Aromatase—a brief overview. Annu Rev Physiol. 2002; 64:93–127. doi: 10.1146/annurev.physiol.64.081601.142703 11826265

[14] Parducz A, Hajszan T, Maclusky NJ, Hoyk Z, Csakvari E, Kurunczi A, et al. Synaptic remodeling induced by gonadal hormones: neuronal plasticity as a mediator of neuroendocrine and behavioral responses to steroids. Neuroscience. 2006; 138(3):977–85. doi: 10.1016/j.neuroscience.2005.07.008 16310961

[15] McEwen BS, Milner TA. Understanding the broad influence of sex hormones and sex differences in the brain. J Neurosci Res. 2017; 95(1–2):24–39. doi: 10.1002/jnr.23809 27870427 PMC5120618

[16] Bachmann G, Bancroft J, Braunstein G, Burger H, Davis S, Dennerstein L, et al. Female androgen insufficiency: the Princeton consensus statement on definition, classification, and assessment. Fertil Steril. 2002; 77(4):660–5. doi: 10.1016/s0015-0282(02)02969-2 11937111

[17] Pastor Z, Holla K, Chmel R. The influence of combined oral contraceptives on female sexual desire: a systematic review. Eur J Contracept Reprod Health Care. 2013; 18(1):27–43. doi: 10.3109/13625187.2012.728643 23320933

[18] Davis SR, Baber R, Panay N, Bitzer J, Perez SC, Islam RM, et al. Global Consensus Position Statement on the Use of Testosterone Therapy for Women. J Clin Endocrinol Metab. 2019; 104(10):4660–6. doi: 10.1210/jc.2019-01603 31498871 PMC6821450

[19] Bosanac SS, Trivedi M, Clark AK, Sivamani RK, Larsen LN. Progestins and acne vulgaris: a review. Dermatol Online J. 2018; 24(5). 30142728

[20] Cheewadhanaraks S, Choksuchat C, Dhanaworavibul K, Liabsuetrakul T. Postoperative depot medroxyprogesterone acetate versus continuous oral contraceptive pills in the treatment of endometriosis-associated pain: a randomized comparative trial. Gynecol Obstet Invest. 2012; 74(2):151–6. doi: 10.1159/000337713 22722530

[21] Jones EE. Androgenic effects of oral contraceptives: implications for patient compliance. Am J Med. 1995; 98(1A):116S–9S. doi: 10.1016/s0002-9343(99)80069-2 7825631

[22] Kahn HS, Curtis KM, Marchbanks PA. Effects of injectable or implantable progestin-only contraceptives on insulin-glucose metabolism and diabetes risk. Diabetes Care. 2003; 26(1):216–25. doi: 10.2337/diacare.26.1.216 12502684

[23] Lortscher D, Admani S, Satur N, Eichenfield LF. Hormonal Contraceptives and Acne: A Retrospective Analysis of 2147 Patients. J Drugs Dermatol. 2016; 15(6):670–4. 27272072

[24] Kim C, Seidel KW, Begier EA, Kwok YS. Diabetes and depot medroxyprogesterone contraception in Navajo women. Arch Intern Med. 2001; 161(14):1766–71. doi: 10.1001/archinte.161.14.1766 11485510

[25] Africander DJ, Storbeck KH, Hapgood JP. A comparative study of the androgenic properties of progesterone and the progestins, medroxyprogesterone acetate (MPA) and norethisterone acetate (NET-A). J Steroid Biochem Mol Biol. 2014; 143:404–15. doi: 10.1016/j.jsbmb.2014.05.007 24861265

[26] Nanba AT, Rege J, Ren J, Auchus RJ, Rainey WE, Turcu AF. 11-Oxygenated C19 Steroids Do Not Decline With Age in Women. J Clin Endocrinol Metab. 2019; 104(7):2615–22. doi: 10.1210/jc.2018-02527 30753518 PMC6525564

[27] Schiffer L, Kempegowda P, Sitch AJ, Adaway JE, Shaheen F, Ebbehoj A, et al. Classic and 11-oxygenated androgens in serum and saliva across adulthood: a cross-sectional study analyzing the impact of age, body mass index, and diurnal and menstrual cycle variation. Eur J Endocrinol. 2023; 188(1). doi: 10.1093/ejendo/lvac017 36651154

[28] Dowsett M, Lal A, Smith IE, Jeffcoate SL. The effects of low and high dose medroxyprogesterone acetate on sex steroids and sex hormone binding globulin in postmenopausal breast cancer patients. Br J Cancer. 1987; 55(3):311–3. doi: 10.1038/bjc.1987.61 2952154 PMC2001763

[29] Segall-Gutierrez P, Du J, Niu C, Ge M, Tilley I, Mizraji K, et al. Effect of subcutaneous depot-medroxyprogesterone acetate (DMPA-SC) on serum androgen markers in normal-weight, obese, and extremely obese women. Contraception. 2012; 86(6):739–45. doi: 10.1016/j.contraception.2012.05.148 22959905

[30] Kovalevsky G, Ballagh SA, Stanczyk FZ, Lee J, Cooper J, Archer DF. Levonorgestrel effects on serum androgens, sex hormone-binding globulin levels, hair shaft diameter, and sexual function. Fertil Steril. 2010; 93(6):1997–2003. doi: 10.1016/j.fertnstert.2008.12.095 19394598

[31] Jain J, Kwan D, Forcier M. Medroxyprogesterone Acetate in Gender-Affirming Therapy for Transwomen: Results From a Retrospective Study. J Clin Endocrinol Metab. 2019; 104(11):5148–56. doi: 10.1210/jc.2018-02253 31127826

[32] Lawrie TA, Hofmeyr GJ, De Jager M, Berk M, Paiker J, Viljoen E. A double-blind randomised placebo controlled trial of postnatal norethisterone enanthate: the effect on postnatal depression and serum hormones. Br J Obstet Gynaecol. 1998; 105(10):1082–90. doi: 10.1111/j.1471-0528.1998.tb09940.x 9800931

[33] Goldman AL, Bhasin S, Wu FCW, Krishna M, Matsumoto AM, Jasuja R. A Reappraisal of Testosterone’s Binding in Circulation: Physiological and Clinical Implications. Endocr Rev. 2017; 38(4):302–24. doi: 10.1210/er.2017-00025 28673039 PMC6287254

[34] Klipping C, Duijkers I, Mawet M, Maillard C, Bastidas A, Jost M, et al. Endocrine and metabolic effects of an oral contraceptive containing estetrol and drospirenone. Contraception. 2021; 103(4):213–21. doi: 10.1016/j.contraception.2021.01.001 33428907

[35] Zimmerman Y, Eijkemans MJ, Coelingh Bennink HJ, Blankenstein MA, Fauser BC. The effect of combined oral contraception on testosterone levels in healthy women: a systematic review and meta-analysis. Hum Reprod Update. 2014; 20(1):76–105. doi: 10.1093/humupd/dmt038 24082040 PMC3845679

[36] Jeppsson S, Gershagen S, Johansson ED, Rannevik G. Plasma levels of medroxyprogesterone acetate (MPA), sex-hormone binding globulin, gonadal steroids, gonadotrophins and prolactin in women during long-term use of depo-MPA (Depo-Provera) as a contraceptive agent. Acta Endocrinol (Copenh). 1982; 99(3):339–43. doi: 10.1530/acta.0.0990339 6461995

[37] Zhao XJ, Shao GX, Zhang J, Sang GW. Effects of steroid contraceptive on serum levels of sex hormone binding globulin in women. Shengzhi Yu Biyun. 1992; 12(4):13–7. 12286664

[38] Vermeulen A, Verdonck L, Kaufman JM. A critical evaluation of simple methods for the estimation of free testosterone in serum. J Clin Endocrinol Metab. 1999; 84(10):3666–72. doi: 10.1210/jcem.84.10.6079 10523012

[39] Avenant C, Bick AJ, Skosana SB, Dlamini S, Balakrishna Y, Moliki JM, et al. Misreporting contraceptive use and the association of peak study progestin levels with weight and BMI among women randomized to the progestin-only injectable contraceptives DMPA-IM and NET-EN. PLoS One. 2023; 18(12):e0295959. 10.1371/journal.pone.0295959 PMC10745193 38134043

[40] Eisenhofer G, Fassnacht M. Steroid Profiling for Adrenocortical Disorders: A Pathway for Omics-Based Diagnostics. Clin Chem. 2017; 63(12):1787–9. doi: 10.1373/clinchem.2017.281048 29018028

[41] Skiba MA, Bell RJ, Islam RM, Handelsman DJ, Desai R, Davis SR. Androgens During the Reproductive Years: What Is Normal for Women? J Clin Endocrinol Metab. 2019; 104(11):5382–92. doi: 10.1210/jc.2019-01357 31390028

[42] Schaffir JA, Isley MM, Woodward M. Oral contraceptives vs injectable progestin in their effect on sexual behavior. Am J Obstet Gynecol. 2010; 203(6):545 e1-5. doi: 10.1016/j.ajog.2010.07.024 20800828

[43] Bloem LM, Storbeck KH, Swart P, du Toit T, Schloms L, Swart AC. Advances in the analytical methodologies: Profiling steroids in familiar pathways-challenging dogmas. J Steroid Biochem Mol Biol. 2015; 153:80–92. doi: 10.1016/j.jsbmb.2015.04.009 25869556

[44] Handelsman DJ, Wartofsky L. Requirement for mass spectrometry sex steroid assays in the Journal of Clinical Endocrinology and Metabolism. J Clin Endocrinol Metab. 2013; 98(10):3971–3. doi: 10.1210/jc.2013-3375 24098015

[45] Moreira IFA, Bianchini MP, Moreira GRC, Almeida AM, Rezende BA. Sexual function and metabolic/hormonal changes in women using long-term hormonal and non-hormonal contraceptives: a pilot study. BMC Womens Health. 2020; 20(1):240. doi: 10.1186/s12905-020-01107-1 33109159 PMC7590685

[46] Africander D, Verhoog N, Hapgood JP. Molecular mechanisms of steroid receptor-mediated actions by synthetic progestins used in HRT and contraception. Steroids. 2011; 76(7):636–52. doi: 10.1016/j.steroids.2011.03.001 21414337

[47] Kemppainen JA, Langley E, Wong CI, Bobseine K, Kelce WR, Wilson EM. Distinguishing androgen receptor agonists and antagonists: distinct mechanisms of activation by medroxyprogesterone acetate and dihydrotestosterone. Mol Endocrinol. 1999; 13(3):440–54. doi: 10.1210/mend.13.3.0255 10077001

[48] Wiegratz I, Kuhl H. Progestogen therapies: differences in clinical effects? Trends Endocrinol Metab. 2004; 15(6):277–85. doi: 10.1016/j.tem.2004.06.006 15358281

[49] Achilles SL, Mhlanga FG, Musara P, Poloyac SM, Chirenje ZM, Hillier SL. Misreporting of contraceptive hormone use in clinical research participants. Contraception. 2018; 97(4):346–53. doi: 10.1016/j.contraception.2017.09.013 28966052 PMC5858917

