# Supplementary material for: The injectable contraceptives depot medroxyprogesterone acetate and norethisterone enanthate substantially and differentially decrease testosterone and sex hormone binding globulin levels: A secondary study from the WHICH randomized clinical trial
Source: PLoS One. 2024 Aug 23;19(8):e0307736. doi: 10.1371/journal.pone.0307736 (PMC11343371; doi:10.1371/journal.pone.0307736)
Supplement: S1 File — (DOCX) [file pone.0307736.s001.docx]

**Supplementary Fig and Tables**

**Table S1. Comprehensive method validation data^*^.**

|  | **Testosterone concentrations validated (ng/mL)** | | | | |
| --- | --- | --- | --- | --- | --- |
|  | **0,05** | **0,1** | **0,5** | **5** | **50** |
| **Matrix effect (%)** | ND | ND | ND | -0.75 | ND |
| **Extraction efficiency (%)** | ND | ND | ND | 103.7 | ND |
| **Accuracy (%BIAS)** | -15.11 | -1.6 | -4.32 | ND | 9.79 |
| **Precision Intra-assay (%CV)** | 7.71 | 6.6 | 0.06 | ND | 2.77 |
| **Precision Inter-assay (%CV)** | 15.4 | ND | 16.0 | 19.4 | 17.4 |

^*^Accuracy, precision, matrix effects and extraction efficiency are shown. Matrix effect, Extraction efficiency, Accuracy and Intra-assay Precision n = 10; Inter-assay precision n = 7. ND – Not determined.

**Table S2.** **Limit of Detection (LOD), lower & upper limit of quantification (LLOQ and ULOQ)*.**

|  | **Testosterone concentration** | |
| --- | --- | --- |
|  | **ng/mL** | **nmol/L** |
| **LOD** | 0.025 | 0.087 |
| **LLOQ** | 0.050 | 0.173 |
| **ULOQ** | 50.00 | 173.37 |

^*^LOD (ng/mL, nmol/L), LLOQ (ng/mL, nmol/L) and ULOQ (ng/mL, nmol/L) were determined as described in the methods.

**Table S3. Total testosterone (nmol/L), SHBG (nmol/L) and free testosterone (pmol/L) outcomes at baseline and 25 weeks for a subgroup (PP analysis).**

|  | **DMPA-IM** | | **NET-EN** | |
| --- | --- | --- | --- | --- |
|  | **Median**  **(IQR)** | **n** | **Median**  **(IQR)** | **n** |
| **Total Testosterone (nmol/L)** | | | | |
| D0 | 0.586  (0.361; 0.836) | 182 | 0.607  (0.402; 0.870) | 149 |
| 25W | 0.416  (0.274; 0.610) | 183 | 0.250  (0.179; 0.376) | 148 |
| Change  (25W - D0) | -0.133  (-0.340; -0.010) |  | -0.333  (-0.576; -0.179) |  |
|  |  | |  | |
| **SHBG (nmol/L)** | | | | |
| D0 | 45.5  (33.6; 70.9) | 183 | 53.2  (36.8; 74.3) | 149 |
| 25W | 31.3  (24.6; 43.3) | 182 | 17.7  (12.25; 22.8) | 148 |
| Change  (25W - D0) | -13.45  (-27.3; -3.00) |  | -35.25  (-55.5; -21.5) |  |
|  |  | |  | |
| **Free Testosterone (pmol/L)** | | | | |
| D0 | 7.18  (2.79; 14.79) | 182 | 6.56  (3.31; 15.28) | 149 |
| 25W | 5.51  (2.31; 11.51) | 182 | 3.83  (1.03; 8.29) | 147 |
| Change  (25W - D0) | -0.79  (-6.21; 1.07) |  | -3.00  (-8.95; 0.80) |  |

IQR (25th and 75th Percentile); Significant differences were detected within (D0-25W) and between contraceptive groups at 25W, with p < 0.0001 for all analyses except for free testosterone, where p = 0.0079 for change within the DMPA-IM arm and p = 0.0079 for differences at 25W between contraceptive arms. p-values were obtained from mixed effects linear regression, accounting for repeated measurements per participant and clustering by site.

B

A

C

**Fig S1. Total testosterone (nmol/L), SHBG (nmol/L) and free testosterone (nmol/L), outcomes at baseline and 25 weeks for a subgroup (PP analysis).** Graphs indicate median with interquartile range (IQR). Significant differences were calculated by mixed effects linear regression, accounting for repeated measurements per participant and clustering by site and are indicated by asterisks where ** and **** represent p<0.01 and p<0.0001, respectively.

**Table S4. Baseline characteristics of women by randomization method for a subgroup (PP analysis)^$^.**

|  | **Baseline (D0)** | | | |
| --- | --- | --- | --- | --- |
|  | **DMPA-IM** | | **NET-EN** | |
|  |  | **n** |  | **n** |
| **Age, years: Mean (SD)** | 25.3 (4.9) | 183 | 25.1 (4.5) | 149 |
| **Ethnicity** |  | 183 |  | 149 |
| Xhosa | 123 (67.2) |  | 99 (66.4) |  |
| Zulu | 55 (30.1) |  | 50 (33.6) |  |
| Mixed race | 1 (0.5) |  | 0 (0.0) |  |
| Other African ethnicity | 4 (2.2) |  | 0 (0.0) |  |
| **Previous use of method^#^** |  |  |  |  |
| DMPA-IM | 139 (76.0) | 183 | 107 (71.8) | 149 |
| NET-EN | 50 (27.3) | 183 | 42 (28.2) | 149 |
| **Marital status** |  | 183 |  | 149 |
| Single | 179 (97.8) |  | 145 (97.3) |  |
| Married | 4 (2.2) |  | 4 (2.7) |  |
| **Highest level of education** |  | 183 |  | 149 |
| Primary school, complete | 1 (0.5) |  | 2 (1.3) |  |
| High school, not complete | 77 (42.1) |  | 49 (32.9) |  |
| High school, complete | 63 (34.4) |  | 65 (43.6) |  |
| Post high school education | 42 (23.0) |  | 33 (22.1) |  |
| **Source of income** |  | 183 |  | 149 |
| Unemployed | 153 (83.6) |  | 124 (83.2) |  |
| Employed | 30 (16.4) |  | 25 (16.8) |  |

^$^Unless indicated otherwise, values represent n-value (%); ^#^Prior to exclusion period, numbers given are for those that responded, and in brackets are % of those that responded.
