# Supplementary material for: The injectable contraceptives depot medroxyprogesterone acetate and norethisterone enanthate substantially and differentially decrease testosterone and sex hormone binding globulin levels: A secondary study from the WHICH randomized clinical trial
Source: PLoS One. 2024 Aug 23;19(8):e0307736. doi: 10.1371/journal.pone.0307736 (PMC11343371; doi:10.1371/journal.pone.0307736)
Supplement: S1 Appendix — (DOCX) [file pone.0307736.s002.docx]

**Supplementary methods: Appendix 1**

**UHPLC-MS/MS testosterone quantification**

Testosterone was quantified by UHPLC-MS/MS on stored samples from 435 WHICH study participants at D0 and 25W.

**Solvents and steroids**

Methyl tert-butyl ether (MTBE), LC-MS grade water, LC-MS grade methanol, LC-MS grade formic acid, ammonium fluoride (NH_4_F), phosphate buffered saline (PBS), bovine serum albumin (BSA) and non-stripped human serum were purchased from Sigma-Aldrich (South Africa). Testosterone was purchased from Sigma-Aldrich (South Africa), while deuterated testosterone‑16,16,17‑D3 (T-d3) used as an internal standard was purchased from Toronto Research Chemicals (North York, ON, Canada). Individual stock solutions of testosterone (1 mg/mL), as well as an internal standard stock solution (containing 0.075 ng/μL T-d3), were prepared in absolute ethanol or acetonitrile, respectively, and stored at -20°C until use. Serum was heat inactivated by incubating at 56°C for 30 mins. Thereafter the heat inactivated serum was stripped twice by incubating in activated charcoal (2.5% (w/v) charcoal, 0.25% (w/v) dextran, 0.1 M Tris-HCL pH 8) for 30 mins at 45°C, centrifuged at 5000 x g for 5 mins to pellet the charcoal and passed through a 0.22 μm filter.

**Calibration curve and internal quality control (IQC) sample preparation**

The 1 mg/mL testosterone stock solution was used to prepare two standard master mixes (1 000 ng/mL and 1 ng/mL) in ethanol. These standard master mixes were subsequently used to prepare a twelve-point calibration curve, which included calibrations at 0, 0.1, 0.025, 0.05, 0.1, 0.25, 0.5, 1, 5, 10, 25 and 50 ng/mL, by addition of the appropriate volume of the standard master mix to surrogate matrix PBS containing 0.1% (w/v) BSA (500 μL final volume). Three independent calibration curves were prepared, extracted and quantified with each batch of serum samples.

IQC samples were prepared in bulk (10 mL) by spiking stripped human serum with the testosterone master mixes (0.05, 0.5, 5 and 50 ng/mL final concentration). Thereafter, each of the four homogenous IQCs was aliquoted (500 μL) and stored at -20°C until use. An aliquot of each IQC was thawed, extracted and quantified with each batch of serum samples.

**Testosterone extraction**

Testosterone was extracted by mixing 500 μL calibration curve standard, IQC or serum samples with 100 mL ultra-pure water containing 0.75 ng T-d3 (10 μL from 0.075 ng/μL stock) in clean disposable glass tubes. Thereafter the samples were loaded onto supported liquid extraction Isolute (SLE+) columns (Biotage, Uppsala, Sweden) and incubated for 5 minutes. Testosterone was eluted into clean disposable glass tubes with 3 × 2 mL MTBE and subsequently dried under a stream of nitrogen gas at 40°C. The glass tubes containing the extracted testosterone was rinsed with 2 mL MTBE and dried again. Testosterone was then carefully resuspended in 100 μL 1:1 methanol:water (v/v) and stored at -20°C prior to analysis.

**Instrument and chromatographic conditions for UHPLC-MS/MS**

Testosterone was separated using an ACQUITY UPLC system (Waters Corporation, Milford, USA) and an ACQUITY UPLC HSS T3 column (2.1 mm x 50 mm, 1.8 μm particle size) (Waters Corporation, Milford, USA), coupled to an ACQUITY UPLC HSS T3 VanGuard Pre-column (2.1 mm X 5 mm, 1.8 µm) (Waters Corporation, Milford, USA). The column temperature was 60°C and the injection volume was 10 μL. The mobile phase consisted of 0.1% (v/v) formic acid in water (Mobile phase A) and methanol (Mobile phase B (MPB)). A gradient inlet method was used to separate the steroids using a constant flow rate of 0.60 mL/min according to the following protocol: 45% MPB from 0-5 min; 75% MPB from 5-5.10 min; 100% MPB from 5.10-6.50 min; 45% MPB from 6.50-7.50 min and hold for re-equilibration.

Post column infusion of NH_4_F [1](3 mM in 50% methanol) at a constant flow rate of 20.0 μL/min using the fluidics system on the mass spectrometer under full software control was performed to enhance the ionization of testosterone. Quantitative mass spectrometric detection was carried out using a Xevo TQ-S triple quadrupole mass spectrometer (Waters, Milford, USA). Testosterone was analyzed in multiple reaction monitoring (MRM) mode using an electrospray probe in the positive ionization mode (ESI+). The following settings were used: capillary voltage of 3.7 kV, desolvation temperature 350°C, desolvation gas 650 L/h and cone gas 150 L/h. MRM transitions for the quantifier and qualifier were T (289.2 🡪 97.2; 289.2 🡪 109) and T-d3 (292.2 🡪 97.1; 292.2 🡪 109.1).

**Extraction efficiency and matrix effects**

We determined the recovery of testosterone (extraction efficiency) by spiking six separate aliquots of surrogate matrix with testosterone (5 ng/mL final) and internal standard mix (0.75 ng T-d3), both before (pre) and after extraction, but before the dry-down step (post). Matrix effects were determined by spiking 6 individual vials of MTBE with testosterone master mix (5 ng/mL final) and internal standard mix (0.75 ng T-d3) before drying down (no). Recovery and matrix effects were then determined as follows:

Recovery (%) = [Concentration (pre)/Concentration (post)] x 100

Matrix effects (%) = [(Concentration (post) – Concentration (no)) / Concentration (no)] x 100

Mean values between 80 - 120% for recovery and between -20 – 20% for matrix effects were considered acceptable (Supplemental table S1).

**Accuracy and precision**

Accuracy was determined by individually spiking 10 aliquots of surrogate matrix with testosterone master mix at four different concentrations (0.05, 0.1, 0.5 and 10 ng/mL final). Thereafter internal standard mix was added, and samples extracted as above. A bias of the observed concentration and the nominal concentration between -20% and +20% was considered acceptable.

Precision was determined by preparing in bulk surrogate matrix spiked with testosterone master mix at four different concentrations (0.05, 0.1, 0.5 and 10 ng/mL final) and internal standard mix. These homogenous samples were then aliquoted (500 μL) and each aliquot (n = 10) extracted and quantified in the same batch to assess intra-assay precision. Inter-assay precision was determined by quantifying the IQC samples in parallel with each batch of serum samples analysed over the course of seven independent UHPLC-MS/MS runs. A coefficient of variation (CV) ≤ 20% was considered acceptable.

**Limit of detection (LOD) and lower limit of quantification (LLOQ)**

The LOD for testosterone was defined as the lowest concentration at which the signal to-noise (S/N) ratio of the quantifier ion was greater than three. The LLOQ for testosterone was defined as the lowest concentration at which: the S/N ratio of the quantifier ion was greater than three; the S/N ration of the qualifier ion was greater than 10; an acceptable precision [CV ≤ 20%] and a bias not greater than ±20%. The upper limit of quantification (ULOQ) was defined as the maximum concentration on the linear region of the calibration curve with an acceptable precision [CV ≤ 20%] and a bias not greater than ±20%.

1. Schiffer L, Shaheen F, Gilligan LC, Storbeck KH, Hawley JM, Keevil BG, et al. Multi-steroid profiling by UHPLC-MS/MS with post-column infusion of ammonium fluoride. J Chromatogr B Analyt Technol Biomed Life Sci. 2022; 1209:123413.
