# Supplementary material for: The injectable contraceptives depot medroxyprogesterone acetate and norethisterone enanthate substantially and differentially decrease testosterone and sex hormone binding globulin levels: A secondary study from the WHICH randomized clinical trial
Source: PLoS One. 2024 Aug 23;19(8):e0307736. doi: 10.1371/journal.pone.0307736 (PMC11343371; doi:10.1371/journal.pone.0307736)
Supplement: Attachment — Submitted filename: Responses to Reviewers.docx [file pone.0307736.s003.docx]

| **Additional Editor Comments:** |  |
| --- | --- |
| Thank you for your submission and please see reviews below. It has been exceedingly difficult to find reviewers so apologize for delay in return of this and in fact there were invitations to over 25 people to act as a reviewer for this manuscript. This is a substudy of measurements of hormones from the larger WHICH study. There is reference to findings of the larger study multiple times and the results of this study are helpful to put context around the data presented here. Unfortunately, the references are designated as "submitted" so the reader is not able to see that study. This led me to wonder if it would be better to publish this one after the publication of the main study. Given the time that has passed, this main study may be slated for publication which would be ideal. | The main study has now been published in PLOS One (PLoS One. 2024 Mar 26;19(3):e0295764). On this revised version, we have cited it. We have also deleted parts of the methods that we had put in the first submission of the current paper, pertaining to the primary paper, and to our progestin paper which is now published in PLOS One (PLoS One. 2023 Dec 22;18(12):e0295959), which are no longer necessary. |
| Lines 54-55 Will the reader understand what the abbreviations D0 and 25W mean? Would it be clearer to say in the beginning of the sentence that there was a substantial decrease from baseline to week 25 (if I am understanding what these abbreviations mean) and then give the % decline in the values.  It appears that the decrease is calculated as 25W-D0 and the decrease is expressed as a negative %. A negative decrease (seems like a double negative) could be interpreted as an increase. If not, would be clearer to say that the decrease is a % (without the negative sign) or that the value at 25W is x% lower than D0? This comment pertains to the results section as well. If this is the standard way this information is expressed, please disregard the comment. | In the last sentence of the previous paragraph we had already defined D0 and 25W. For further clarity we have revised this sentence, now on lines 49-52 (tracked version), by adding the phrase “post initiation”. We have also revised the part of this sentence relating to pairs of samples, which was queried elsewhere, so the sentence now reads:  “We measured testosterone by UHPLC-MS/MS and SHBG by immunoassay in matched pairs of serum samples collected at baseline (D0) and at peak serum progestin levels at 25 weeks post initiation (25W) (n = 214-218 pairs)”.  Regarding the negative sign for percentages: We have left the values in the text for decreased percentages, when stated in brackets, as negative, which is how they are presented in the tables. This is conventional, to indicate a decrease in the percentage. We think putting a value in brackets in a sentence, that is not in the same conventional format as in the table, is confusing. However, we agree that when we report the values without brackets, we should not use the negative before percentage as in for example: “DMPA-IM and NET-EN significantly reduced median SHBG levels from baseline to 25W by 7% and 66%, respectively |
| Line 59-The % difference (decrease) for testosterone and SHBG is expressed without a leading negative sign. Why is the difference expressed as a positive number here and negative above? Is there a reason why both a decrease in median level and a mean percentage difference is given? | In lines 54-59 (tracked version) we indicated a decrease in the percentage difference from baseline to 25W for each individual contraceptive arm as shown in Table 3. In lines 59-62 (tracked version), which refers to data in Table 4, this is a different calculation and measure. This is the percentage difference in the change for one contraceptive arm compared to the change in the other contraceptive arm. We have added a description of the calculation as a footnote in Table 4. |
| Line 64-The time period of measurement expressed here appears to be D0 to 24W whereas above it is D0 and 25W. Is this correct? Why are these times different and why the is the formatting different for the presentation of the number of weeks (24W vs W25)? | Thank you for pointing this out. It should read 25W and has been changed accordingly. |
| Line 119- Is the implication here that decreased levels of testosterone impact susceptibility to HIV though a decreased libido that leads to less exposure or is it another mechanism? Should this be explained further here? | Thank you for this useful comment. Yes, that is what is proposed. We have inserted the sentence (now lines 120-121): “Thus, decreased testosterone levels may decrease libido resulting in less exposure to HIV.” |
| Line 152-It might be interesting to tell the reader whether it is the bound or the free testosterone that is active (or the different activities of these 2 moieties), if there is room, especially as it is later stated how the different hormones impact, free, bound and total testosterone. | Thank you for the suggestion. While we have shortened parts of this paragraph, we have inserted now the following sentence (now line 159-162): “Total testosterone includes both the biologically inactive, circulating, SHBG-bound testosterone, as well as the biologically active testosterone circulating either free (not bound to plasma protein) or that weakly bound to albumin [33].” |
| Line 188-Presumably this is an open label study due the difference in the dosing interval of the agents. It would be helpful to state this. | Line 173 in the methods (now line 186) already stated: “This study is an ancillary study from the open-label randomized WHICH clinical trial.” We therefore do not think it is necessary to mention this again. |
| Line 196-Are the PrEP drugs referred to here for HIV? That should be stated. | Correct. We have modified to state: “pre-exposure prophylaxis drugs (for HIV)” |
| Line 258-It is stated that a subgroup was used for the PP analysis and that this group excluded of persons who used “non-study progestin >1.5”. It is very unclear what this is and is it possible that examples be given? | We have elaborated more on exclusions in this paragraph and included examples (currently paragraph line 298). The work on non-study progestins is now published in PLOS One (PLoS One. 2023 Dec 22;18(12):e0295959). |
| Line 298-Why are the numbers 219 and 217 listed in the white lines of this table under “previous use of method” whereas in the other parts of this table these totals are in the gray lines?  “Previous use of method” should be reworded to “Previous method used”.  Since the numbers for this category add to greater than the total for each arm, need to tell the reader that some participants used both of the methods listed.  Why are Single and Married listed under this heading? | The numbers 219 and 217 should have been listed as totals (grey lines) corresponding to “Previous use of method”. We have now corrected this, thank you.  In terms of rewording, our phrase more accurately describes what question was asked of the participants i.e. whether they had used DMPA-IM and/or NET-EN prior to the exclusion period for contraceptive use. The suggested phrase “Previous method used” is a different question and would mean one would need to list what other methods were used, which was not asked for and not recorded.  In terms of the numbers not adding up, the numbers are correct. Some individuals could have used both methods (at different times) prior to the exclusion period We have added a footnote to the Table to clarify this as follows: “Note that some participants reported using both contraceptive methods prior to the exclusion period.”  Re Single and Married-Thank you for pointing out that Single and Married were incorrectly listed under “Previous method used”. This was a mistake that has been corrected. |
| Line 306-Usually, the p value is listed after the 2 values being compared. | We have moved the p-value to after the 2 values being compared. |
| Line 342-Perhaps state for clarity “DMPA-IM and NET-EN significantly reduced median SHBG levels from baseline to 25W by -27% and -66%, respectively (Table 3)".  Is there a reason that the levels are presented in an inconsistent manner here? For example, the SHBG levels are presented as values in nmol/L with a difference expressed in concentration in nmol/L but the SHBG is presented as a negative % with the difference presented as % change. | Re previous line 342: We have revised the values in that paragraph based on the requested new statistical analysis. We now have a sentence (currently lines 417-419) that reads: “DMPA-IM and NET-EN significantly reduced SHBG levels from D0 to 25W by 29.8% and 65.1%, respectively (p < 0.0001 for both arms) (Tables 2 and 3).”  We use the standard units (i.e. nmol/L for SHBG) when we report the actual values observed at baseline or 25 weeks (for both arms), as well as when we compare absolute values at one timepoint (i.e. 25 weeks) between the two arms in Table 2. However, in Table 3, when we report the change from baseline to 25 weeks of the one contraceptive arm relative to that of the other contraceptive arm, we use percentage values to better illustrate the magnitude of one change compared to the other. |
| Line 347-“Median free testosterone concentrations at baseline and 25W were not significantly different between the two arms at baseline (p = 0.842)” Does this mean that all 4 of the measurements were not different from each other? Or does that mean there was not a difference between baseline and 25W in each of the 2 arms? Please clarify the statement. The statement seems to contradict the next sentence of “At 25W the 349 median free testosterone level in the DMPA-IM arm of 5.00 pmol/L was significantly 350 higher than 4.00 pmol/L in the NET-EN arm (p = 0.0124)”. This entire paragraph is hard to followed and needs to be rewritten for clarity. | Thank you for pointing this out. There was a mistake in the original sentence. We have corrected it as follows (now lines 423-424):  “Median free testosterone concentrations at baseline were not significantly different between the two arms (p = 0.9867) (Fig 2 and Table 2).”  In addition, we have also edited the rest of the paragraph to improve clarity. |
| Line-358-Does data need to be presented for both medians and means? If so why? Since it is stated that results were similar for medians and means, it is not clear if this information is needed. | We have removed the mean data originally given in Supplementary Table S3. We agree that they are not necessary. We have also removed reference to the means previously in that table, from the text. |
| Line 374-The discussion seems a bit long and could be shortened with the discussion on other studies made more terse. | The discussion has been substantially shortened. |
| Line 376-It is stated “significant decreases from D0 to 25W in measured total testosterone (-24% and -54%)”. The decrease is a percent, not a negative percent. By saying that there is a decrease from baseline to 25W, it is understood that the value is lower, the % expresses the magnitude of the decrease. Adding the negative sign seems to make this confusing. | See response to one of the editor’s comments above. |
| Line 431-Does 4/5 and 3/5 mentioned here refer to markers of sexual behavior? If so, perhaps reword to “no significant differences were detected for 4/5 markers in 432 the DMPA-IM arm and for 3/5 markers in the NET-EN arm”. | We have taken out most of the detailed discussion on sexual behavior as this is presented in the primary paper which is now published (PLoS One. 2024 Mar 26;19(3):e0295764). Thus, this sentence is now deleted. |
| Line 437-Sometimes HIV is used and sometimes HIV-1 is used, as here. Is there a reason for this difference? | Thank you. We have changed to HIV throughout. |
| Line 438-Data on sexual desire is not included in this paper and the discussion should be focused on the data from this study. That there was a difference in sexual desire is noted in the WHICH cohort is mentioned on Line 429 and that seems sufficient and the discussion here should focus on this paper’s data, especially since other paper is not yet published. | We have taken out the detailed discussion on sexual behavior from the discussion. |
| Line 444-The statistical power of the WHICH study is not the subject of this discussion and no information has been given to the reader to support claims about the power of the study on sexual behavior results. | We have removed this sentence. |
| Line 460-Can it be stated that data have been reported when the paper has not yet been published? | The paper has now been published (PLoS One. 2024 Mar 26;19(3):e0295764) and we have included the reference |
| Line 481-What is the basis for saying that the study is high-powered? No information on the power of the study is given in the methods.  How could there be misreporting of DMPA-IM or NET-EN use when these are the interventions that are being examined in this study and presumably being administered in a controlled manner? | We have removed use of the word high-powered throughout. Power studies are reported in the primary paper, but we feel it is sufficient in this paper to reference the primary paper, and to note the fact that the study is randomized and to give the number of participants.  It is common for women to lie about not using treatments prior to initiation, within the exclusion period. It is also common for them to take other contraceptives that they are not assigned to during the trial eg COCs while on an injectable (to control bleeding). There is a lot of literature on this which we cover in our progestin paper. We have inserted a sentence now (lines 565-567) in the revised manuscript that reads: “We and others have reported that it is common for women to not self-report non-study progestin use before initiation and during clinical trials on contraception (39, 49)”. |
| Line 490-stated “we would have had to exclude a large proportion of the women (for use of non-study progestins) and substantially reduced the power of this study” What are these and how many of the participants used them? Was it possible to exclude such women from the study? If not, why not? | We have added more detail on this point in the Methods. Since our non-study progestin paper is now published (PLoS One. 2023 Dec 22;18(12):e0295959), we do not think it is necessary or appropriate to include more detail on this topic in this paper, especially since we have been asked to shorten the discussion. |
| Line 506-“Our findings of increased risky sexual behavior for DMPA-IM relative to NET-EN users” no data on this has been presented and this should not be part of the conclusions of this study. Further is stated that it is likely that progestins are determinants of androgenic effects.  Given that it is also stated that a large number of the women in this study may have used non-study progestins, does this introduce significant bias? | We have changed the sentence to read:  “Our published findings of increased risky sexual behavior for DMPA-IM relative to NET-EN users are consistent with the differential decrease in total endogenous testosterone levels.” The primary paper has been published in PLOS One, and we reference it now (PLoS One. 2024 Mar 26;19(3):e0295764). The sentence refers to how the testosterone values in the submitted paper relate to our published findings, which we think is valid for concluding on the implications of the testosterone results in the submitted paper.  Regarding significant bias, we show in the current paper that this does not appear to be the case for the testosterone and SHBG data, from our PP analysis. We have elaborated further by stating on page 30 (lines 571-575 tracked version): “These PP analyses resulted in the same significant differences compared to the mITT results, and comparable median values for total and free testosterone and SHBG, suggesting that non-study progestin use did not introduce significant bias to these values.”  Regarding bias in the sexual behavior data, this is possible, but we do not think it is appropriate to speculate on that data in the current paper, especially as we have been asked to shorten the manuscript. |
| **Reviewer #1: General comments:** |  |
| This manuscript reports a secondary analysis from a clinical trial that explored the differences in serum testosterone and sex hormone binding globulin levels between two injectable contraceptives.  My main concern regards the statistical methods. The methods seem to lack coherence. For instance, I found it confusing why the authors claim non-parametric tests are required for all data, but then use methods that assume normality. There may also be methods used that are not reported in the methods section.  Also, I will point out that the choice of running a statistical model is more about whether the residuals are normally distributed and not the outcome itself. Linear models are somewhat robust against deviations from normality. Looking at figure 2, I think analyzing a log-transformed outcomes is feasible. I would much prefer if you were able to fit a single statistical model for each of outcome and then evaluate differences between groups and time points. That would reduce the risk of type I errors and allow for much more coherent methods and results sections.  Although I am here to evaluate design and methods, I strongly suggest reducing the introduction and probably the discussion. In the vast majority of papers, the introduction can be 3-4 paragraphs, i.e., (1) broad background on problem, (2) specifics on problem to be addressed, and (3) objectives and brief synopsis of design and methods.  Thus, I don't feel the paper presently meets criterion 3 (Experiments, statistics, and other analyses are performed to a high technical standard and are described in sufficient detail) and criterion 5 (The article is presented in an intelligible fashion and is written in standard English) since it's wordy. Though, I think you could fix these. | Thank you for your suggestion which has improved the coherence and rigor of the results. We have now presented results using a single statistical model for each outcome.  All statistical methods are reported in the Methods section.  We have shortened the Introduction and Discussion. |
| **Specific comments:** |  |
| 1. (lines 51-52) If you are using paired tests, this should be the sample size of the pairs. | We were referring to number of pairs i.e. number of participants with paired samples. These varied very slightly between arms, and also between which analyte was reported, due to technical reasons e.g. one sample out of around 200 samples may have been spilled or not read properly by the machine. We do not think it is appropriate to give this level of detail in the abstract. We have revised the sentence in the abstract to read as follows: “We measured testosterone by UHPLC-MS/MS and SHBG by immunoassay in matched pairs of serum samples collected at baseline (D0) and at peak serum progestin levels at 25 weeks post initiation (25W) (n = 214-218 pairs)”. In addition, we have given the actual number of samples for which we obtained data for each analyte, in the Methods. These numbers were and are still in Table 2. We have also added a footnote on this to Table 2 and the Supp Table S4. |
| 2. (lines 54-60) Confidence intervals or p-values are needed for all of these comparisons. | Thank you for your comment. We have now added p-values for these comparisons in the abstract. |
| 3. (lines 225-226) This must be described somewhere in the manuscript especially since the information has not been published. There is no word count for PLOS ONE, so you should feel free to include this information here. | We have removed the sentences “Study and non-study progestins were similarly measured by UHPLC-MS/MS….have been submitted elsewhere.”. This paper has now been published (PLoS One. 2023 Dec 22;18(12):e0295959). We now give more clarity on this and reference the published paper in the Data analysis section. |
| 4. In lines 267-269, you note that all data are not normally distributed but then in lines 274-277 you use a model that assumes normality. Granted, you log-transformed your outcomes for the mixed models. Why can't that be done for the simple tests as well? | Thank you for your suggestion. The presentation of the original data as median values (with IQRs) is more useful to researchers in the field as compared to the log-transformed values. All p-values in the manuscript have now been calculated using a single model for each outcome. |
| 5. (Table 1, line 295, line 306, …) Claiming "similarity" is incorrect when using superiority tests. These statements suggest that the absence of evidence indicates evidence of absence. You can claim no difference, but not similarity. Statements like this need to be revised since a p>0.05 doesn't necessarily mean there were no differences. Significance testing for baseline imbalance in randomized trials been has regarded as unnecessary (see Altman DG. Comparability of randomised groups. The Statistician 1985; 125-136; Senn, S. Testing for baseline balance in clinical trials. Statistics in Medicine 1994; 1715-1726). I recommend removing the significance testing entirely from table 1. | We appreciate the reviewer's recommendation and agree that the inclusion of significance testing for baseline characteristics needs to be carefully considered. However, we feel its inclusion in Table 1 is necessary. This study presents a subgroup of the original cohort, which necessitates a thorough examination of baseline characteristics to ensure the validity and reliability of the comparison. Readers may appreciate the transparency of having this information readily available to assess the baseline comparability and generalizability of the study findings. |
| 6. (Table 3, line 315) I don't recall something in the methods describing analyses of percentage change. | Thank you for your comment. We have adjusted our analyses. The new percentage changes presented have all been calculated via the back-transformation of the model coefficients. |
| 7. (Table 4) I encourage you to report your effects on the scale in which they are analyzed. | Thank you for your comment. The results from Table 4 are the back-transformed coefficients from the linear model using log-transformed data resulting in it being reported as percentage change. The caption indicates that mean percentages are presented. |
| 8. In addition, you use the word "similar" in other situations, e.g., lines 363 and 366. Similar is vague and subjective and I suggest being specific about the comparisons made in these statements. | Thank you for this comment. We have removed the use of the word “similar” where it is inappropriate. |
| Reviewer #2: |  |
| thanks for the opportunity to review this well written paper  A few minor comments only | Thank you very much for this comment. |
| Line 103 – please clarify what you mean by the “6 month injection” as neither of these are 6 month injections. | We have clarified this in the Methods where we have the following revised sentence:  “Blood samples were collected at baseline (D0) and at 25 weeks (25W), i.e. about 7 days after the 24-week (24W) NET-EN (the 4^th^ NET-EN injection) or DMPA-IM (the 3^rd^ DMPA-IM) injection, and serum was separated and stored at -80^◦^C.” |
| Please explain line 105 further | See above. In addition, the primary paper is now published (PLoS One. 2024 Mar 26;19(3):e0295764) so any additional details will be published by the time the current paper is accepted. |
| Line 196 – assume you mean PrEP for HIV - clarify | Correct. We have modified to state: “pre-exposure prophylaxis drugs (for HIV)” |
