# Supplementary material for: The injectable contraceptives depot medroxyprogesterone acetate and norethisterone enanthate substantially and differentially decrease testosterone and sex hormone binding globulin levels: A secondary study from the WHICH randomized clinical trial
Source: PLoS One. 2024 Aug 23;19(8):e0307736. doi: 10.1371/journal.pone.0307736 (PMC11343371; doi:10.1371/journal.pone.0307736)
Supplement: Attachment — Submitted filename: Responses to Reviewers.docx [file pone.0307736.s004.docx]

Responses to Reviewers

| Significance testing in table 1:  The main point of my comment is to provide a more accurate assessment of baseline imbalance. It is not about transparency. For example, two groups with small sample sizes will likely have p>0.05 for all characteristics. So, then having a smaller sample size is better…? No, it's because p-values are a poor assessment of baseline comparability since the p-value is driven by the sample size. Other methods, such as standardized mean differences, provide a better assessment of differences. I still believe this should be changed. | Thank you for your comment. After consideration, we have opted to remove the p-values from Table 1, as per your initial suggestion. Appropriate changes have been made to the manuscript text. |
| --- | --- |
| Normality assumption:  I'm fine with reporting medians and IQRs for summary statistics, but in Table 2 you are reporting medians and IQRs right next to p-values from regression models that assume normality. This remains confusing for me and I think has the potential for misinterpretation. If someone takes a quick glance at this, I fear s/he will either (1) think the summary statistics are means and 95% CIs or, more likely, think the p-value are for a difference in medians. I encourage you to report what is actually being tested next to the p-value to avoid this potential for confusion. | Thank you for your comment. Since the presentation the medians and IQRs are of interest and primarily for descriptive purposes, we have opted to remove the p-values from Table 2. The data being tested that corresponds to the p-values are presented in Tables 3 and 4. Appropriate changes have been made to the manuscript text. |
| Please provide details on the back-transformation mentioned in line 258 since this is reported in the text and a table. | Thank you for your suggestion. We have added the following text to the data analysis section: ‘A model coefficient, $\beta$, on the log-scale can be back-transformed using $e^{\beta}$. To facilitate the interpretation of the results, we report the percentage changes on the original scale, calculated as $\left( e^{\beta}-1 \right)\times100\%$.’ |
| (Table 3) For free testosterone, why is the DMPA-IM vs. NET-EN p-value < 0.05 when the two 95% CIs overlap? | Thank you for your comment. We have checked the data and the confidence interval is correct.  95% confidence intervals for the mean may overlap but the p-value for the difference can be statistically significant. Briefly, the 95% confidence intervals presented in Table 3 provide the range of plausible values for the population mean. When testing for a significant difference, the confidence interval of the differences is assessed (whether the interval contains 0, indicating no difference, or not) rather than the confidence intervals for each group. Therefore, confidence intervals can overlap even when the difference between groups is statistically significant, as the overlap reflects the inherent variability and sample size rather than the precise statistical difference between means or proportions.  Austin & Hux (2002) provides further details on overlapping confidence intervals.  Austin, P.C. and Hux, J.E. (2002) ‘A brief note on overlapping confidence intervals’, Journal of Vascular Surgery, 36(1), pp. 194–195. doi:10.1067/mva.2002.125015. |
| (lines 299-301 and Table 4) It's not clear to me what the values in this table mean. For total testosterone, is the -2.9 a difference at baseline? Or is it a mean percentage change? It's not clear from the title or the notes what these values are. | Thank you for your comment. We have edited Table 4 to include more detailed headings. |
